# Dietary Agents in Cancer Chemoprevention and Treatment

**DOI:** 10.1155/2012/749310

**Published:** 2012-12-18

**Authors:** Julian J. Raffoul, Omer Kucuk, Fazlul H. Sarkar, Gilda G. Hillman

**Affiliations:** ^1^Department of Medicine, Emory University School of Medicine, Atlanta, GA 30322, USA; ^2^Department of Hematology and Medical Oncology, Winship Cancer Institute, Emory University School of Medicine, Atlanta, GA 30322, USA; ^3^Department of Pathology, Barbara Ann Karmanos Cancer Institute, Wayne State University, Detroit, MI 48201, USA; ^4^Department of Radiation Oncology, Barbara Ann Karmanos Cancer Institute, Wayne State University School of Medicine, Detroit, MI 48201, USA

Cancer chemoprevention using natural or synthetic compounds to prevent or suppress the development of cancer, is an area of active investigation. Many compounds belonging to diverse chemical classes have been identified as potential chemopreventive agents, including vitamins and minerals, naturally occurring phytochemicals, and synthetic compounds. Understanding the molecular mechanisms of cancer chemoprevention is not only important for the safe application of these compounds in populations of patients at high risk for cancer, but also allows for further development of novel treatment regimens for cancer patients.

This special issue contains original research as well as review articles that are intended to stimulate the continuing efforts to understand the use of dietary agents in cancer chemoprevention and treatment. The lead article by S. N. Saldanha and T. O. Tollefsbol provides a comprehensive review of dietary agents that have shown strong chemopreventive and therapeutic properties *in vitro*. They also discuss the design and modification of these bioactive compounds for pre-clinical and clinical applications. 

Dietary intake of foods rich in antioxidant compounds has been suggested to be cancer protective. However, randomized clinical trials and epidemiologic studies on the association between intake of foods rich in antioxidants and cancer incidence have yielded mixed results. M. Y. Wei and E. L. Giovannuci discuss the epidemiologic considerations of lycopene as a chemopreventive agent, including measurement of lycopene, its major source in the diet, and the assessment of prostate cancer incidence and progression, with particular emphasis on the effect of PSA screening on this association. K. Zhou and J. J. Raffoul discuss the composition and cancer-protective effects of major phenolic antioxidants in grape skin and grape seed extracts. M. A. Parasramka and S. V. Gupta provide original research demonstrating the anticancer properties of garcinol alone, or combined with curcumin, on pancreatic cancer cells. Garcinol, a polyisoprentylated benzophenone extracted from the rind of the fruit *Garcinia indica*, a plant found in tropical regions, has antioxidant and anti-inflammatory properties and its role as anticancer agent is thoroughly discussed in the review from N. Saadat and S. V. Gupta. 

Two manuscripts discussing the effect of dietary agents on DNA repair capacity are also part of this special issue. In a manuscript by J. J. Raffoul et al., the potential for targeting the DNA base excision repair enzyme APE1/Ref-1 using dietary agents such as soy isoflavones, resveratrol, curcumin, ascorbate, and alpha-tocopherol is discussed. The potential for these natural compounds to be combined with chemotherapy or radiotherapy for the more effective treatment of cancer are also reviewed. A proposed mechanism of action is discussed and an attempt is made to delineate which of the two activities of APE1/Ref-1 (DNA repair versus redox activation of cellular transcription factors) is responsible for the observed effects. The second manuscript by R. Rosati et al. reviews the role for dietary folate in the prevention of colorectal cancer. Data are presented which demonstrate that inhibition of DNA repair is protective in the development of preneoplastic colon lesions, both when folate is depleted and when it is not. This manuscript is a comprehensive review of the literature and provides a critical analysis of the experimental designs used in folate and colorectal cancer research. 

Two additional manuscripts detailing the ability of dietary agents to sensitize cancer cells to chemotherapy and radiotherapy are included in this special issue. An original research article by S. Duangmano et al. demonstrates that curcurbitacin B, a plant phytochemical, inhibited breast cancer cell proliferation in a dose-dependent manner and caused radiosensitization of human breast cancer cells via G2/M cell cycle arrest. Furthermore, an original research article by K. Sahin et al. demonstrate that genistein, a soy isoflavone, sensitizes cervical cancer cells to cisplatin via inhibition of NF-kappa B and Akt/mTOR cell signaling pathways.

This special issue concludes with a report of a clinical study demonstrating the prevention of anthracycline-induced cardiac toxicity through supplementation with selenium in a group of pediatric cancer patients. 

Research efforts aimed at understanding the role of dietary agents and phytochemicals in cancer prevention and treatment are likely to yield high-impact results that have the potential for immediate clinical applications. Furthermore, combination of phytochemicals and nutritional agents with therapies for advanced cancers, including radiotherapy and chemotherapy, would benefit from a complementary and safe approach using dietary agents to mitigate the adverse effects of these therapies on normal tissues while enhancing the therapeutic efficacy. Elucidation of the mechanisms of interaction between dietary agents and conventional cancer treatments will have a major impact on understanding the molecular mechanisms of cancer chemoprevention and will ultimately result in clinical use of dietary agents as an adjunct to standard cancer treatment.

*Julian J. Raffoul* *Julian J. Raffoul* *Omer Kucuk* *Omer Kucuk* *Fazlul H. Sarkar* *Fazlul H. Sarkar* *Gilda G. Hillman* *Gilda G. Hillman*

